# Influenza a virus NS1 resembles a TRAF3-interacting motif to target the RNA sensing-TRAF3-type I IFN axis and impair antiviral innate immunity

**DOI:** 10.1186/s12929-021-00764-0

**Published:** 2021-10-05

**Authors:** Chun-Yang Lin, Meng-Cen Shih, Hung-Chun Chang, Kuan-Jung Lin, Lin-Fang Chen, Sheng-Wen Huang, Mei-Lin Yang, Sheng-Kai Ma, Ai-Li Shiau, Jen-Ren Wang, Kuan-Ru Chen, Pin Ling

**Affiliations:** 1 Department of Microbiology and Immunology, College of Medicine, National Cheng Kung University, 70101 Tainan, Taiwan; 2 Department of Medical Laboratory Science and Biotechnology, College of Medicine, National Cheng Kung University, 70101 Tainan, Taiwan; 3 National Mosquito-Borne Diseases Control Research Center, National Health Research, 70101 Tainan, Taiwan

**Keywords:** Influenza virus, NS1, RIG-I, TLR3, TLR7, Interferon, TRAF3

## Abstract

**Background:**

Influenza A virus (IAV) evolves strategies to counteract the host antiviral defense for establishing infection. The influenza A virus (IAV) non-structural protein 1 (NS1) is a key viral factor shown to counteract type I IFN antiviral response mainly through targeting RIG-I signaling. Growing evidence suggests that viral RNA sensors RIG-I, TLR3, and TLR7 function to detect IAV RNA in different cell types to induce type I IFN antiviral response to IAV infection. Yet, it remains unclear if IAV NS1 can exploit a common mechanism to counteract these RNA sensing pathways to type I IFN production at once, then promoting viral propagation in the host.

**Methods:**

Luciferase reporter assays were conducted to determine the effect of NS1 and its mutants on the RIG-I and TLR3 pathways to the activation of the IFN-β and NF-κB promoters. Coimmunoprecipitation and confocal microscopic analyses were used to the interaction and colocalization between NS1 and TRAF3. Ubiquitination assays were performed to study the effect of NS1 and its mutants on TRAF3 ubiquitination. A recombinant mutant virus carrying NS1 E152A/E153A mutations was generated by reverse genetics for biochemical, ex vivo, and in vivo analyses to explore the importance of NS1 E152/E153 residues in targeting the RNA sensing-TRAF3-type I IFN axis and IAV pathogenicity.

**Results:**

Here we report that NS1 subverts the RIG-I, TLR3, and TLR7 pathways to type I IFN production through targeting TRAF3 E3 ubiquitin ligase. NS1 harbors a conserved FTEE motif (a.a. 150-153), in which the E152/E153 residues are critical for binding TRAF3 to block TRAF3 ubiquitination and type I IFN production by these RNA sensing pathways. A recombinant mutant virus carrying NS1 E152A/E153A mutations induces higher type I IFN production ex vivo and in vivo, and exhibits the attenuated phenotype in infected mice, indicating the importance of E152/E153 residues in IAV pathogenicity.

**Conclusions:**

Together our work uncovers a novel mechanism of IAV NS1-mediated immune evasion to promote viral infection through targeting the RNA sensing-TRAF3-type I IFN axis.

**Supplementary Information:**

The online version contains supplementary material available at 10.1186/s12929-021-00764-0.

## Background

Influenza A virus (IAV) is a major pathogen causing respiratory tract diseases, and constantly poses a threat to public health by seasonal epidemics or sporadic pandemics [42]. IAV exploits diverse mechanisms to subvert the innate immune responses, particularly type I interferon (IFN) production, to successfully establish infection [14]. Particularly, high pathogenic IAV strains may even derail the host innate immune responses to cause a cytokine storm, in which inflammatory cytokines are excessively produced whereas the type I IFN production is suppressed [21, 26]. The mechanisms underlying IAV-mediated evasion and dysregulation of host innate immunity to affect the pathogenesis remain elusive. Type I IFNs act as a major driver to trigger antiviral innate immunity at the early infection via inducing hundreds of interferon-stimulated genes (ISGs) that function to restrict viral propagation in host cells at the different steps of the viral life cycle [15, 38, 49]. Furthermore, type I IFNs link to the induction of the adaptive immune responses that mediate a broad spectrum of antiviral immunity leading to viral clearance [38].

Three key RNA sensors, including RIG-I, TLR3, and TLR7, are implicated in detecting IAV infection in different cell types to trigger type I IFNs and inflammatory cytokines [8]. RIG-I is a cytosolic RNA sensor acting in fibroblasts, tracheal epithelial cells, and conventional dendritic cells (cDCs) to detect the 5’-triphosphate signature of influenza single-stranded RNA (ssRNA) genome to induce type I IFN production [9, 24, 28, 35, 43]. TLR7 plays a key role in plasmacytoid dendritic cells (pDCs) to recognize the IAV ssRNA gene segments in endosomes to trigger type I IFNs [10, 28, 36]. Endosomal TLR3 is shown to detect IAV infection in lung epithelial cells to elicit the antiviral immune response [16, 31]. Notably, animal studies suggest that the RIG-I and TLR7 pathways cooperate to defend against IAV infection in vivo [44], while TLR3 may play a pathological role in triggering excessive inflammation and tissue damage during acute IAV infection [30]. Interestingly, a recent study in human patients revealed that TLR3 deficiency in children underscores IAV-mediated pneumonitis [32], suggesting a protective role for TLR3 in humans during natural IAV infection. Upon detecting IAV RNA molecules, RIG-I engages with mitochondrial adaptor MAVS to trigger downstream signaling while endosomal TLR3 and TLR7 activate downstream signaling via two TIR domain-containing adaptors Trif and MyD88, respectively. These RNA sensor-adaptor complexes further link to two common signaling axes which are mediated by two E3 ubiquitin ligases TRAF3 and TRAF6 [50]. For instance, activated MAVS binds to TRAF3 directly, leading to the K63-linked ubiquitination and activation of TRAF3, which subsequently activates the TBK1-IRF3 axis for type I IFN induction. Meanwhile, activated MAVS promotes the K63-linked ubiquitination and activation of TRAF6 for the activation of the IKK-NF-κB axis and then the production of proinflammatory cytokines. TRAF3 and TRAF6 are shown to exert similar roles in the TLR3 and TLR7 pathways [50].

The nonstructural protein 1 (NS1) of IAV is a key viral factor shown to promote viral spread through regulating viral RNA processing and mediating immune evasion [2, 25, 29]. Notably, the amino acid residues R38 and K41 of NS1 are essential for binding viral RNA and also play a critical role in the downregulation of type I IFNs [11]. Further evidence indicates that NS1 exerts a critical role in counteracting the RIG-I-type I IFN axis and targeting some ISGs like OAS and PKR [2, 25, 29]. NS1 utilizes its N-terminal RNA-binding domain (RBD) to block RIG-I-mediated detection of 5’ triphosphate viral RNA or directly bind to the RIG-I CARD domain [22, 43]. The RNA-binding ability of NS1 also mediates its countermeasure via interaction with other host restriction factors like DDX21 and DHX30 helicases [4, 5]. NS1 is shown to target E3 ubiquitin ligases, TRIM-25, and Riplet, leading to the suppression of RIG-I ubiquitination and activation [12, 13, 27, 46]. However, considering the importance of TLR3 and TLR7 in inducing type I IFN-mediated antiviral responses to IAV infection, we were interested in investigating if IAV NS1 may target the TLR3 and TLR7 pathways or exploit a common mechanism to counteract three IAV RNA sensing pathways to type I IFN production, thus resulting in a greater advantage for IAV survival in the host.

## Methods

### Cell

HEK293, HEK293T, and A549 cells were described previously [3, 6]. Madin-Darby canine kidney cells (MDCK) were maintained in DMEM supplemented with 7% cosmic calf serum (CCS, Hyclone). To prepare bone marrow-derived dendritic cells (BMDCs), bone marrow cells isolated from 8-12-week C57BL/6 mice were cultured in RPMI 1640 containing 10% fetal bovine serum, GM-CSF 20 ng/ml (PeproTech), and 1% penicillin and streptomycin. These cultured BMDCs were cultured on an uncoated plate and replaced with fresh medium at days 3 and 6.

### Reverse genetics of influenza virus

Influenza A/PR/8 viruses were prepared as previously described [6]. The A/Puerto Rico/8/1934 virus used in this study was generated using an eight-plasmid reverse genetics system. The plasmids were kindly provided by Webster [18]. To generate the recombinant viruses, eight plasmids were cotransfected into the mixed culture of 293T and MDCK cells (9:1 ratio) by Lipofectamine 2000. At 24 h post-transfection, the medium was replaced by DMEM contain 0.3% BSA without serum. At 72 h post-transfection, the supernatant was collected and amplified in MDCK cells. The parental A/Puerto Rico/8/1934 virus and the mutant viruses encoding NS1 E152A/E153A were propagated in 13-day-old, specific pathogen-free embryonated eggs (Animal Health Research Institute, Tamsui, Taiwan). The virus titer was measured by plaque assay on MDCK cells.

### Mice

C57BL/6 (B6) mice were obtained from the National Laboratory Animal Center, Taiwan. All animal protocols were approved by the Institutional Animal Care and User Committee (NCKU-IACUC-103146, NCKU-IACUC-104082, and NCKU-IACUC-103146) at National Cheng Kung University, and all animal experiments were performed by the approved guidelines and regulations.

### Coimmunoprecipitation (Co-IP) and Western blot (WB) analysis

HEK293T cells were transfected with the indicated plasmids. At 24 h post-transfection, coimmunoprecipitation and Western blot analyses were conducted as described previously [3, 6]. Antibodies used for Co-IP and WB were listed in Additional file 1: Table S2.

### Reagents

Poly(I-C) (#tlrl-pic), and 5′-triphosphate double-strand RNA (5′-ppp dsRNA) (#tlrl-3prna) were purchased from InvivoGen.

### Plasmids

hTLR3-FLAG, FLAG-RIG-I, ΔRIG-I, His-MAVS, IFN-β-Luc, pRL-TK, Flag-TRIF, pcDNA6-Myc-His, and IKKi-K38A were described previously [3, 6]. pSGFP2- C1 (Addgene plasmid 22881) was developed by Dorus Gadella. pEBG (Addgene plasmid 22227) was developed by David Baltimore. IKKβ-K44M (Addgene plasmid 11104) was developed by A. Rao (Harvard University). pRK5-HA-K63-Ubiquitin (Addgene plasmid 17606) was developed by Ted Dawson (Johns Hopkins University School of Medicine). Other plasmids were kindly provided as follows: Flag-TRAF3 and Flag-TRAF6 by Karin, M. (Department of Pharmacology, School of Medicine, University of California San Diego) [17]. Flag-TRAF3 mutants by Carl F. Ware (La Jolla Institute for Allergy and Immunology, CA). pHW2000-PR8-PB1, pHW2000-PR8-PB2, pHW2000-PR8-PA, pHW2000-PR8-HA, pHW2000-PR8-NP, pHW2000-PR8-NA, pHW2000-PR8-NS, and pHW2000-PR8-M1 by Robert G. Webster (St. Jude Children’s Research Hospital, Memphis, TN) [18]. 3X-Flag-RIG-I-CARD (amino acid 1-228) and 3X-Flag-MAVS were amplified by PCR and cloned into a p3xFLAG-Myc-CMV-26 vector (Sigma). RIG-I-CARD (amino acids 2-228) was amplified by PCR and cloned into a pEBG vector. GFP-MAVS was amplified by PCR and cloned into a psGFP2-C1 vector. The full-length NS segment of influenza A/PR/8 (PR8) strain was amplified by PCR and then cloned into pcDNA6.0/Myc-His (Invitrogen) to generate an expression construct NS1- Myc-His with a C-terminal Myc-His tag. Similarly, the NS1- RBD- Myc-His (amino acids 1–73) and NS1- ED- Myc-His (amino acids 74–230) were generated by PCR cloning. NS1- ED-C- Myc-His (amino acids 125–230), NS1-1-124- Myc-His (amino acids 1–124), NS1- E152A/E153A- Myc-His, and pHW2000-PR8-NS- E152A/E153A were generated by site direct mutagenesis. Primers used for cloning were listed in Additional file 1: Table S1.

### Luciferase reporter assay

HEK293 cells were cotransfected with a luciferase reporter plasmid (e.g. IFN-β-Luc or ELAM-Luc) and indicated expression constructs. A Renilla luciferase-expressing plasmid was used as an internal control to normalize transfection efficiency. An empty vector (pcDNA6.0/Myc-His) was used to equalize the total amount of plasmids. After 16-24 h post-transfection or indicated stimulation, cells were then lysed with passive lysis buffer (Promega). The luciferase activities in lysates were measured by the Dual-Luciferase assay (Promega) according to the manufacturer’s protocol.

### In vivo ubiquitination assays

HEK293T cells were transfected as indicated. N-ethylmaleimide (10 mM) were added to the RIPA lysis buffer (0.5% Deoxycholate, 1% Triton X-100, 25 mM Tris (pH = 7.5), 150 mM NaCl, 1mM EDTA, 0.1% SDS) with protease inhibitors cocktail (Sigma), and lysates were cleared by centrifugation. For immunoprecipitation, 1 mg of lysates were incubated with the indicated antibodies for 1 h at 4℃, 1% Triton X-100, protein G beads (Pierce) for another 1 h at 4 ℃. The beads were washed 4 times with RIPA lysis buffer (with protease inhibitors) and then boiled with 4× SDS containing sample buffer at 75 ℃ for 5 min to elute the proteins.

### Plaque assay

The standard plaque assay was performed to determine the influenza virus titer. Briefly, the 6-well plate was seeded with approximately 6 × 10^5^ MDCK cells per well for at least 16 h to allow cell attachment. Before virus infection, the cell monolayer was washed with 1× DPBS and 2× DMEM, respectively. Each well was incubated with 200 µl and the serial tenfold diluted virus-containing samples for 1 h incubation. The plates were shaken every 15 min during the incubation period. After 1 h, the virus-containing supernatants were removed and the cell was washed by 1× dPBS to remove the unattached virus. Then, the cell monolayer was added 2 ml of 1.6% sterile agarose mixed with equal volume 2× DMEM and was incubated at 37 ℃ for 72 h. When the plaques were formed, the DMEM-containing agarose was removed and the cells were fixed and stained with 4% paraformaldehyde and crystal violet for 30 min. Then the plates were washed and air-dried at room temperature. Then the plaque numbers were counted in duplicate and the viral titer was calculated.

### Confocal microscopy

For immunofluorescence, cells were fixed with 4% paraformaldehyde (PFA) in PBS for 15 min followed by permeabilization with 0.2 % Triton X-100 for 10 min. Cells were washed with PBS (pH 7.2-7.4) and blocked with 0.1% BSA in PBST. Anti-TRAF3 antibody (sc-948, Santa Cruz); 1:50, anti-Myc antibody (clone 4A6, Millipore); 1:1000, Secondary fluorophore-conjugated secondary antibody (Alexa Fluor488 and 564) was obtained from Abcam and used at 1:500 in PBS 0.1% BSA. The nucleus was revealed by 4′,6-diamidino-2-phenylindole (DAPI) staining. The confocal micrographs represent a single optical section through the plane of the cell. Images were acquired with FV10-ASW on an FV1000 inverted microscope (OLYMPUS, Japan) with a 60X, PLAPO, NA: 1.4, WD: 0.15, Oil disc lens. Antibodies used for immunofluorescence were listed in Additional file 1: Table S2.

### RNA isolation and reverse transcription-polymerase chain reaction (RT-PCR)

Total RNAs were isolated by using RNAzol according to the manufacturer’s protocol. cDNA was prepared by using a high-capacity cDNA reverse transcription kit (Applied Biosystems).

### Interaction of NS1 with viral RNA

HEK293T cells in a 10-cm dish were transfected with 15 µg of pcDNA-NS1- Myc-His, pcDNA-NS1-RBD- Myc-His, or pcDNA-NS1-ED- Myc-His plasmids using the Lipofectamine 2000. After 24 h post-transfection, cells were lysed in RIPA buffer (Tris 50mM, NaCl 150 mM, 0.5% sodium deoxycholate, 1% Triton × 100, 0.1% SDS) with protease inhibitor. Insoluble material was removed by centrifuging at 20,000*g* for 10 min. Lysates were purified using Nickle beads (GE Healthcare Life Sciences) in the base buffer (20 mM Hepes pH 7.9, 2 mM EDTA, 15% Glycerol and 0.05% NP40, 50 mM NaCl, 10 µg/ml each of aprotinin and pepstatin, 0.5 mM PMSF, 2 mM DTT and 1× ProtectRNA RNase Inhibitor (Sigma) for 1 h at 4 ℃. The beads were washed with base buffer and incubated with 10 µg of total RNA from IAV-infected HEK293 cells for another 1 h at 4 ℃ to pulldown RNA. Then, bound RNA was sequentially isolated from the beads by the RNAzol method. Isolated RNA was subjected to RT-PCR to determine the amount of viral RNA associated with NS1 and its mutants.

### Enzyme-linked immunosorbent assay (ELISA)

Cytokines were measured using ELISA for supernatants obtained from IAV-infected cells or lung homogenates. ELISA kits were as follows: mouse IL-6 (eBioscience), mouse RANTES (R&D Systems), human IFN-α (Invitrogen), mouse IFN-α, and mouse IFN-β (InvivoGen Lumikine XpressTM mIFN-β). Values represent the mean ± standard error of the mean (SEM) of duplicated samples. Data are representative of two or three experiments.

### Propagation of IAV in cell culture and embryonated chicken eggs

The influenza A/PR/8/34 wild-type virus and IAV carrying NS1 E152A/E153A mutations were generated using an eight-plasmid reverse genetics system. To generate recombinant IAV, plasmids encoding NS WT (pHW2000-PR8-NS) and NS E152A/E153A mutant (pHW2000-PR8-NS E152A/E153A) were co-transfected with other seven expression plasmids (pHW2000-PR8-PB1, pHW2000-PR8-PB2, pHW2000-PR8-PA, pHW2000-PR8-HA, pHW2000-PR8-NP, pHW2000-PR8-NA, and pHW2000-PR8-M1) into co-cultured 293T/MDCK cells into 60 mm dish by using Lipofectamine^TM^ 2000 (Invitrogen). After 24 h of transfection, the medium was replaced by 2 ml of DMEM with 0.3% BSA containing 1 µg/ml of TPCK-Trypsin. At 72 h post-infection, the supernatants were collected and purified by centrifuging at 13,000 rpm for 2 min at 4 °C [18, 37]. Wild-type virus (IAV/PR8) or NS1 E152A/E153A virus (IAV/PR8) were further propagated in 10-days-old, specific pathogen-free (SPF) embryonated chicken eggs. Now the infected eggs were incubated at 35 °C and ~ 60% humidity for 48 h. All procedures were performed under sterile conditions. Before harvesting the allantoic fluid, the chicken eggs were incubated overnight at 4 °C for the coagulation of the embryo’s blood leading it to death. Then the allantoic fluid was centrifuged at 500 g for 5 min at 4 °C and transferred (without taking pellet) to a fresh microtube. Ultimately, the virus-treated allantoic fluid was stocked at a -80 °C freezer for long-term storage and usage.

### IAV infection in mice

C57BL/6 mice on average 6-7 weeks old were anesthetized by intraperitoneal injection of mixture Zoletil 50 and Rompun. Anesthetized mice were infected intranasally with 20 µl of serum-free DMEM medium containing wild-type virus (IAV/PR8) or NS1 E152A/E153A virus (IAV/PR8). Body weights of mice were monitored daily for 14 days and weight loss was determined using two-way ANOVA statistical analysis. Mice that lost 15% of their body weight were considered to have reached human endpoints, and were sacrificed according to the study protocol. Log-rank (Mantel-Cox) test was used for statistical analysis and then used Kaplan-Meier curves to generate the possible survival curves. Pulmonary viral titers of two different genotype mice were analyzed by the Mann-Whitney test.

### IAV growth kinetic

MDCK cells were infected with IAV-WT and with IAV NS1 E152A/E153A mutant viruses at MOI of 0.01 in 24-well. The supernatants were collected at the indicated time points (24, 48, and 72 h) for determining infectious virus particles by plaque assays on MDCK cells.

### Statistical significance

Statistical analyses were performed using unpaired, two-tailed, Student’s t-tests available in GraphPad Prism software (La Jolla, CA, USA). For survival data, we used Kaplan-Meier analysis available in GraphPad Prism software (La Jolla, CA, USA). For weight loss data, we used Two-way ANOVA available in GraphPad Prism software (La Jolla, CA, USA). The P-value below 0.05 was statistically significant.

## Results

### The NS1 effector domain counteracts RIG-I signaling to type I IFN induction via an RNA binding-independent manner

**Fig. 1:**
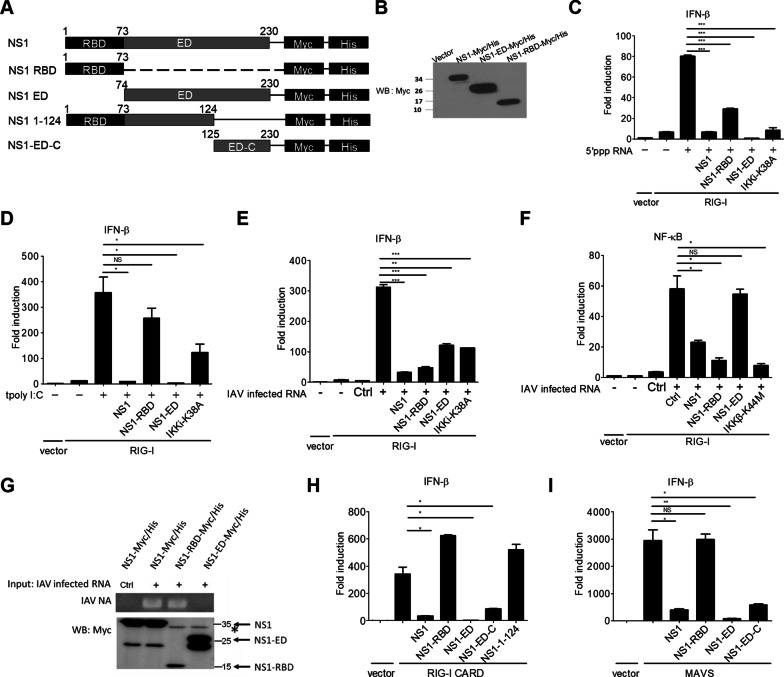
The effect of IAV NS1 and its truncated mutants on blocking RIG-I signaling to IFN-β activation. **A** Schematic structures of IAV NS1 and its truncated mutants with C-terminal tagged Myc/His. **B** The expression of NS1 constructs in HEK293T cells was examined by Western blot (WB) using anti-Myc antibody. **C, D** HEK293 cells were transfected with an IFN-β luciferase reporter (IFN-β-Luc) together with a control vector or RIG-I in combination with NS1, NS1 truncated mutants, or an IKKi mutant as indicated. After 24 h, transfected cells were left untreated or treated with transfection of 5’-triphosphate (5’ppp RNA) (1 µg/ml) (**C**) or poly I:C (0.1 µg/ml) shown as tpoly I:C (**D**). After another 16 h, treated cells were harvested for analyzing the IFN-β promoter activity. **E, F** Similar to the panel C approach, HEK293 cells were transfected with IFN-β-Luc (**E**) or NF-κB luciferase reporter (pELAM-Luc) (**F**) together with a control vector or RIG-I in combination with different NS1 constructs, IKKi-K38A (**E**) or IKKβ-K44M (**F**) as indicated. Transfected cells were left untreated or treated with Control RNA (ctrl, 0.1 µg/ml) and IAV-infected RNA (0.1 µg/ml) transfection, and then were harvested for analyzing the IFN-β (**E**) or NF-κB (**F**) promoter activity. **G** IAV NS1 and its truncated mutants from transfected HEK293T cells were pulled down with nickel beads. The pull-down beads were then incubated with control RNA and IAV-infected RNA, and bound RNA was subjected for PCR amplification for IAV NA RNA (top). The pull-down NS1 and its mutants were examined by WB (bottom). * indicated the non-specific band recognized by the anti-Myc antibody. **H**, **I** HEK293 cells were transfected with IFN-β-Luc together with RIG-I CARD (**H**) or MAVS (**I**) in combination with NS1 and NS1 truncated mutants as indicated. After 16 h, the cells were lysed and analyzed for the IFN-β promoter activity. Values represent the mean ± SD of duplicate samples (**C**-**F**, **H**, and **I**). *P < 0.05, **P < 0. 05, ***P < 0.0005, and NS stands for “not significant” (Student’s *t*-test). Data are representative of three experiments

First, we attempted to explore if IAV NS1 employs other mechanisms to subvert RIG-I signaling to type I IFN-mediated antiviral responses. NS1 was dissected into two truncated mutants, the RNA binding domain (a.a. 1-73) designated NS1-RBD and the effector domain (a.a. 74-230) designated NS1-ED, for assessing their effects on RIG-I signaling to type I IFN induction (Fig. 1A). NS1-RBD and NS1-ED were used for the luciferase reporter assay after their expression was confirmed by immunoblotting (Fig. 1B). HEK293 cells are known to express low or undetectable RIG-I and other innate immune sensors [7, 19, 20], and are commonly used for reconstitution experiments to study innate immune signaling. Ectopic expression of full-length NS1 and NS1-RBD, like IKKi-K38A mutant, impaired RIG-I-mediated IFN-β promoter activation upon 5’-triphosphate RNA stimulation (Fig. 1C). Notably, NS1-ED also showed a significant blocking effect (Fig. 1C). Additionally, NS1 and NS1-ED effectively blocked poly I:C-induced RIG-I signaling to the IFN-β promoter while NS1-RBD only slightly reduced this induction (Fig. 1D). Further experiments using IAV infected RNA indicated that NS1 and NS1-RBD blocked RIG-I signaling to the IFN-β and NF-κB promoters while NS1-ED selectively impaired the activation of the IFN-β promoter but not the NF-κB promoter (Fig. 1E, F). As shown in our previous work [3], a kinase-defective IKKi-K38A mutant was used as a control to show the blocking effect on RIG-I signaling to the IFN-β promoter (Fig. 1D, E). Likewise, a kinase-defective IKKβ-K44M mutant was a control to block RIG-I signaling to the NF-κB promoter (Fig. 1F). These data together suggest that NS1-RBD impairs RIG-I signaling to the IFN-β and NF-κB pathways while NS1-ED primarily targets the RIG-I-IFN-β axis via an RNA binding-independent mechanism.

To test this idea further, we first attempted to exclude a possibility that NS1-ED might contain a cryptic site for viral RNA binding. We investigated the ability of NS1, NS1-RBD, and NS1-ED to bind IAV RNA by an in vitro RNA binding assay. As shown in Fig. 1G, His-tagged NS1 and NS1-RBD were shown to precipitate IAV-NA viral RNA very well. Yet, His-tagged NS1-ED failed to pull down detectable IAV-NA RNA (Fig. 1G). Having confirmed that NS1-ED did not bind IAV RNA, we determined the underlying mechanism by which NS1-ED antagonizes RIG-I signaling to type I IFN activation. To that end, a RIG-I active mutant called RIG-I CARD, which contains the N-terminal two tandem CARDs of RIG-I to trigger downstream signaling without RNA ligand stimulation, was used for our study. RIG-I CARD-induced IFN-β promoter activation was substantially downregulated in the presence of NS1 and NS1-ED but not NS1-RBD (Fig. 1H). To further map the critical region of NS1 responsible for this regulation, we generated two other truncated mutants: the C-terminal region for NS1-ED designated NS1-ED-C (a. a. 125-230) and the RBD plus the N-terminal region of NS1-ED designated NS1-1-124 (a. a. 1-124) for our experiments (Fig. 1A). NS1-ED-C was shown to block RIG-I-CARD-induced IFN-β promoter activation while NS1-1-124 showed no blocking effect (Fig. 1H and Additional file 1: Fig. S1A). The expression of these NS1 truncated mutants was confirmed by immunoblotting (Additional file 1: Fig. S1B). Following this line, we assessed the effect of NS1 and its truncated mutants on the RIG-I downstream adaptor MAVS signaling to the IFN-β promoter. Likewise, MAVS-induced IFN-β promoter activation was downregulated in the presence of NS1, NS1-ED, and NS1-ED-C but not NS1-RBD (Fig. 1I). Collectively, our data reveal a novel NS1-mediated immune evasion via its distal C-terminal effector domain to target the RIG-I-IFN-β axis.

### IAV NS1 interacts with TRAF3 via its effector domain

**Fig. 2:**
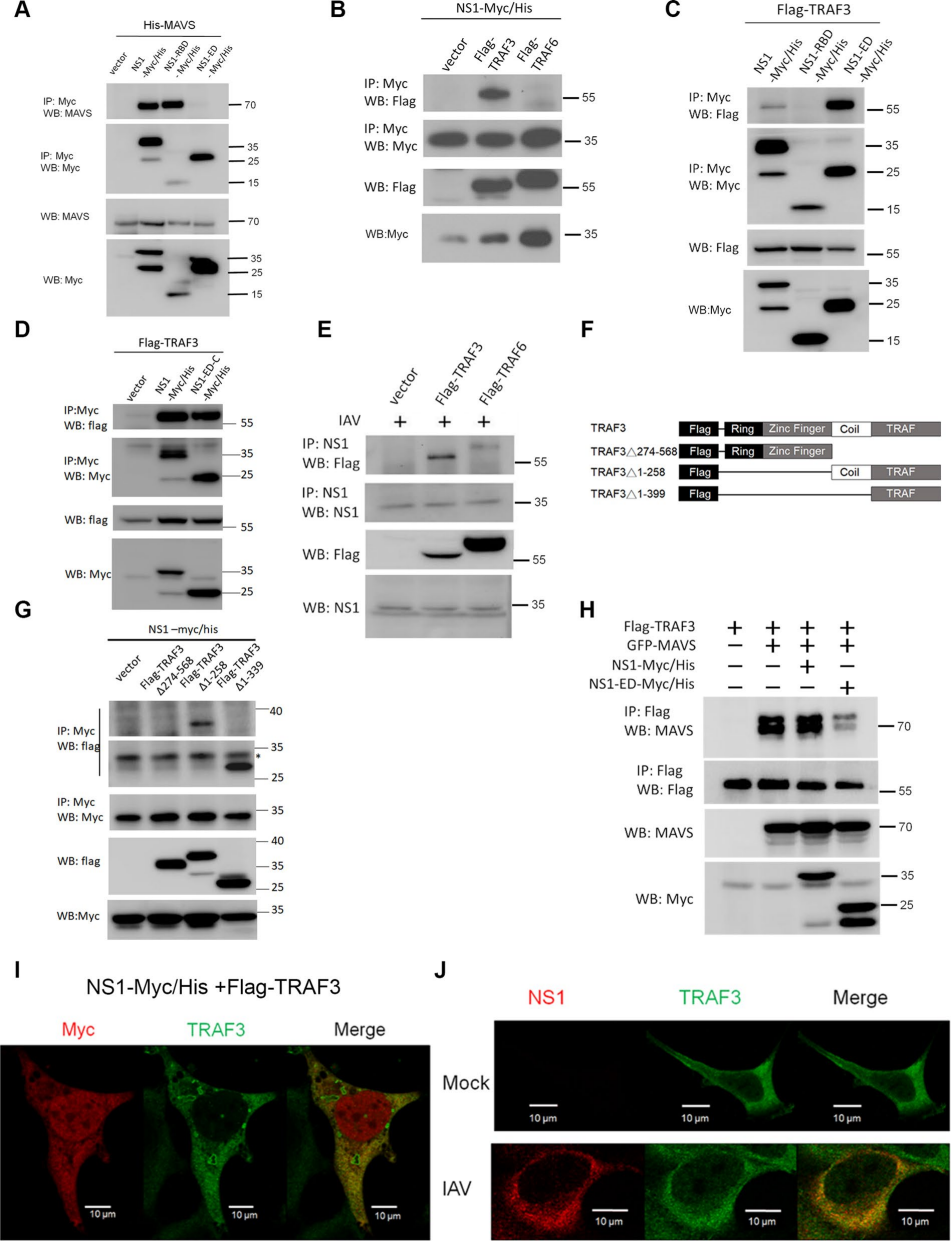
Interaction and colocalization of NS1 and TRAF3 in mammalian cells. **A** HEK293T cells were transfected with His-MAVS together with different NS1-Myc/His constructs. Cell lysates were subjected to the immunoprecipitation (IP)- Western blot (WB) analysis. **B** Like panel **A**, the IP-WB analysis was conducted to examine the interaction of NS1-Myc/His and Flag-TRAF3 or Flag-TRAF6 in HEK293 T cells. **C**,** D** Similarly, the IP-WB analysis was used to map the domain of NS1 critical for binding TRAF3 by transfection of Flag-TRAF3 with NS1-Myc/His or different NS1 truncated mutants as indicated. **E** HEK293T cells transfected with Flag-TRAF3 or Flag-TRAF6 were infected with IAV (PR8, 3 MOI) to examine the interaction of NS1and TRAF3 under virus infection by the IP-WB analysis. **F** Schematic structures of TRAF3 and its truncated mutants with an N-terminal Flag-tag. **G** A similar IP-WB analysis was conducted to determine the domain of TRAF3 for binding NS1 by transfection of NS1-Myc/His with Flag-TRAF3 or its truncated mutants as indicated. * means the non-specific band. **H** HEK293T cells were transfected with Flag-TRAF3 alone or in combination with GFP-tagged MAVS, NS1-Myc/His, or NS1-ED-Myc/His as indicated. Then, the cells were harvested for the IP- WB analysis to assess the effect of NS1 and NS1-ED on the complex formation between MAVS and TRAF3. **I** Confocal microscopy was performed to examine the colocalization of NS1 and TRAF3 in mammalian cells. HEK293 cells were transfected with NS1-Myc/His together with Flag-TRAF3, and the cells were then subjected to the immunofluorescence analysis using anti-Myc and anti-TRAF3 antibodies. **J** Like panel **I**, HEK293 cells transfected with Flag-RIG-I were left untreated or infected with IAV (PR8, 5 MOI) for 16 h. Then the cells were subjected to confocal microscopy using anti-NS1 and anti-TRAF3 antibodies

To gain mechanistic insights into NS1-ED-mediated subversion of the RIG-I-IFN-β axis, we next explored if NS1-ED targets downstream mediators in the RIG-I pathway. Co-immunoprecipitation (Co-IP) was performed to assess the potential interactions between NS1 and several RIG-I downstream mediators, including MAVS, TRAF3, and TRAF6. Our Co-IP results first showed that immunoprecipitation of NS1 and NS1-RBD, but not NS1-ED, could pull down MAVS from HEK293T cells (Fig. 2A), suggesting that MAVS is not the target of NS1-ED. In light of Fig. 1I data, this NS1-RBD-MAVS interaction is likely to have no effect on MAVS signaling to the IFN-β promoter activation. Further, NS1 was shown to preferentially interact with TRAF3 but not TRAF6 in HEK293T cells (Fig. 2B), and both NS1-ED and NS1-ED-C were shown to bind TRAF3 (Fig. 2C, D), suggesting that the C-terminal region of NS1 is essential for binding to TRAF3. Also, the interaction between NS1 and TRAF3 was confirmed in the context of IAV infection (Fig. 2E). Collectively, these results suggest that IAV NS1 binds to TRAF3 via its effector domain, particularly the C-terminal region.

We next investigated the domain of TRAF3 critical for IAV NS1 interaction. Several Flag-tagged TRAF3 deletion mutants, including TRAF3Δ274-568, TRAF3Δ1-258, and TRAF3Δ1-399, were utilized for the Co-IP analysis (Fig. 2F). Our results revealed that immunoprecipitated NS1 was associated with TRAF3Δ1-258 and TRAF3Δ1-399 but not TRAF3Δ274-568, suggesting that the C-terminal TRAF domain of TRAF3 is critical for binding NS1 (Fig. 2G). Notably, the TRAF domain of TRAF3 is responsible for the direct interaction with MAVS, which contains a TRAF3-interaction motif (TIM) (a.a., 455-458), during RIG-I signaling to type I IFN activation [41, 48]. Thus, we further assessed if NS1 and NS1-ED might compete with MAVS to bind TRAF3 in mammalian cells. Our Co-IP results showed that the MAVS-TRAF3 interaction was diminished in the presence of NS1-ED (Fig. 2H). Surprisingly, the full-length NS1 did not reduce the MAVS-TRAF3 interaction in the Co-IP experiment (Fig. 2H). Given that NS1 dampened MAVS signaling to the IFN-β activation (Fig. 1I), together our Co-IP data in Fig. 2 suggest that NS1 interrupts the direct MAVS-TRAF3 interaction to form a trimolecular MAVS-NS1-TRAF3 complex via its RBD and ED, respectively, leading to the disruption of RIG-I signaling.

Confocal microscopy was used to examine the subcellular distribution of IAV NS1 and TRAF3 in mammalian cells. Myc-tagged NS1 and Flag-tagged TRAF3 were co-transfected into HEK293 cells for immunofluorescence (IF) analyses. Our data showed that NS1 was highly co-localized with TRAF3 in the cytoplasm (Fig. 2I). The specificity of immunofluorescent signals was confirmed by immunostaining with secondary antibodies alone (Additional file 1: Fig. S2A). Likewise, the colocalization of NS1 and TRAF3 was confirmed by immunostaining of endogenous TRAF3 and exogenous Myc-NS1 in HEK293 cells (Additional file 1: Fig. S2B). This spatial relationship was also confirmed in the context of IAV infection. The NS1 signal was only detected in HEK293 cells after IAV infection and was highly merged with endogenous TRAF3 (Fig. 2J). Together, our confocal microscopic data revealed that IAV NS1 was colocalized with TRAF3 in the cytoplasm.

### The NS1 effector domain is critical for blocking the ubiquitination and activation of TRAF3 during RIG-I signaling

**Fig. 3:**
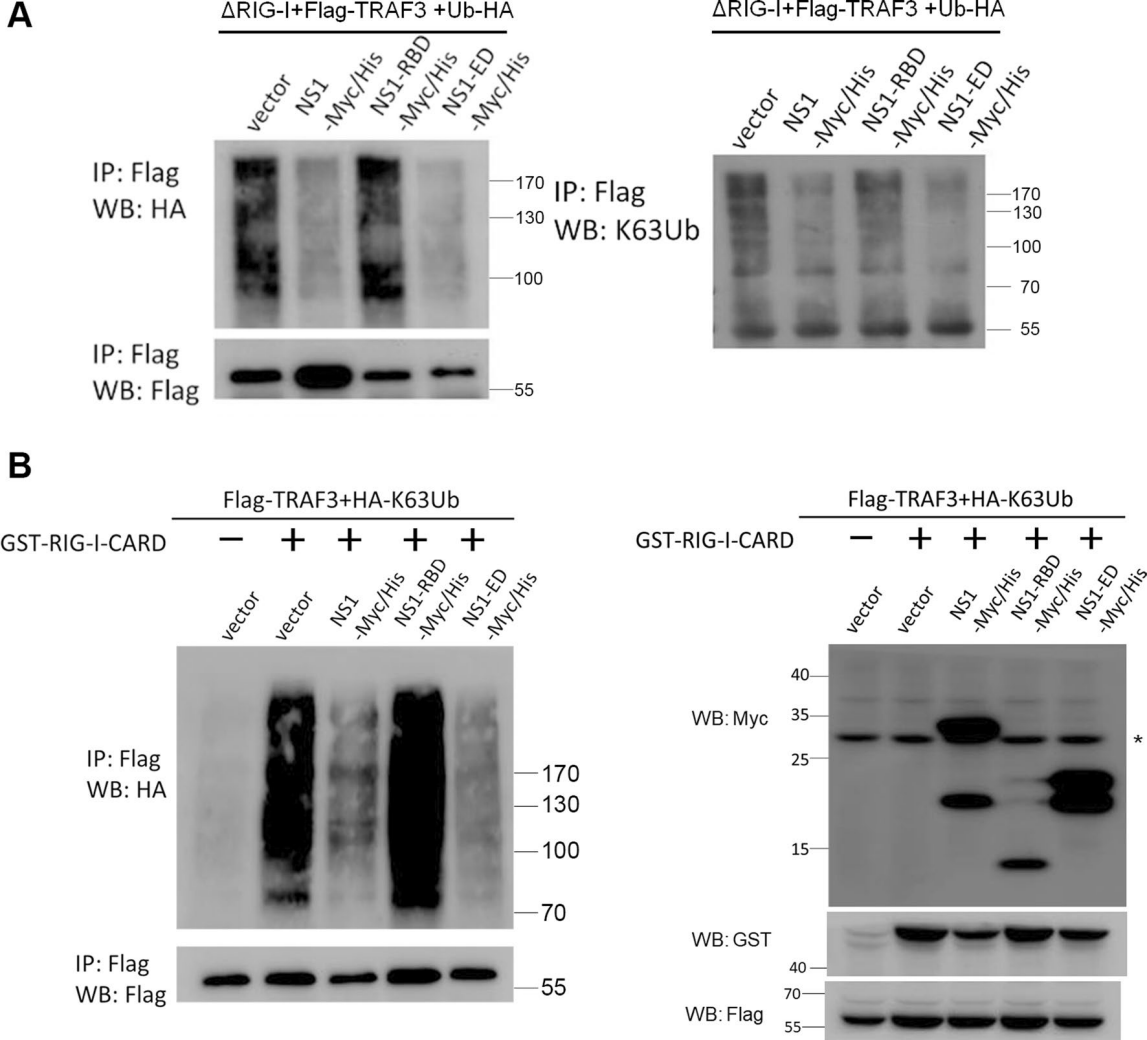
IAV NS1 uses its effector domain to block the K63-linked ubiquitination of TRAF3 during RIG-I signaling. **A** HEK293T cells were co-transfected with HA-tagged Ubiquitin (Ub), Flag-TRAF3, and delta RIG-I (ΔRIG-I) together with different NS1 constructs as indicated. Cell lysates were first immunoprecipitated by anti-Flag antibody and then immunoblotted by anti-HA antibody (left panel) or anti-K63-linked ubiquitin antibody (right panel). **B** HEK293T cells were transfected with HA-tagged K63 Ub, Flag-TRAF3, and GST-RIG-I-CARD together with different NS1 constructs as indicated. A similar IP- WB analysis was used to determine the effect of NS1 and its mutants on the K63-linked ubiquitination of TRAF3 during RIG-I signaling (left panel). Expression of Flag-TRAF3, GST-RIG-I-CARD, or NS1 constructs was confirmed by Western blot analyses using anti-Myc, anti-GST, or anti-Flag antibody as indicated (right panel). * indicated the non-specific band recognized by anti-Myc antibody

TRAF3 is an E3 ubiquitin ligase essential for linking the RIG-I-MAVS axis to type I IFN production [39, 41, 48]. The complex formation of MAVS-TRAF3 facilitates the K63-linked ubiquitination and activation of TRAF3, which in turn activates the TBK1-IRF3 axis for type I IFN production. Having shown that IAV NS1-ED targeted TRAF3 to disrupt the MAVS-TRAF3 complex (Fig. 2H), we were prompted to examine the effect of NS1, NS1-RBD, and NS1-ED on TRAF3 ubiquitination during RIG-I signaling. Our IP-Western blot (WB) analyses showed that immunoprecipitated Flag-TRAF3 was associated with substantial HA-ubiquitin signals after the expression of a RIG-I active mutant ΔRIG-I (Fig. 3A, the left panel). Notably, ΔRIG-I-induced TRAF3 ubiquitination was inhibited by the co-expression of NS1 and NS1-ED but not NS1-RBD (Fig. 3A, the left panel). A similar result was observed using a K63 ubiquitin-specific antibody (Fig. 3A, the right panel). Following this finding, we performed further IP-WB analyses using HA-K63-ubiquitin and another RIG-I active mutant GST-RIG-I-CARD for our study. Our results showed that the K63-linked ubiquitination of TRAF3 induced by RIG-I-CARD was reduced in the presence of NS1 and NS1-ED but not NS1-RBD (Fig. 3B, the left panel). The expression levels of these transfected constructs were confirmed by immunoblotting, including GST-RIG-I-CARD, Flag-TRAF3, and various NS1 constructs with Myc/His-tag (Fig. 3B, the right panel). Further, this K63-linked ubiquitination of TRAF3 was downregulated by NS1-ED-C but not NS1-1-124 (Additional file 1: Fig. S3). These data together suggest that NS1 targets TRAF3 via its effector domain to block the K63-linked ubiquitination of TRAF3 during RIG-I signaling.

### IAV NS1 E152/E153 residues are critical for targeting TRAF3 during RIG-I signaling

**Fig. 4:**
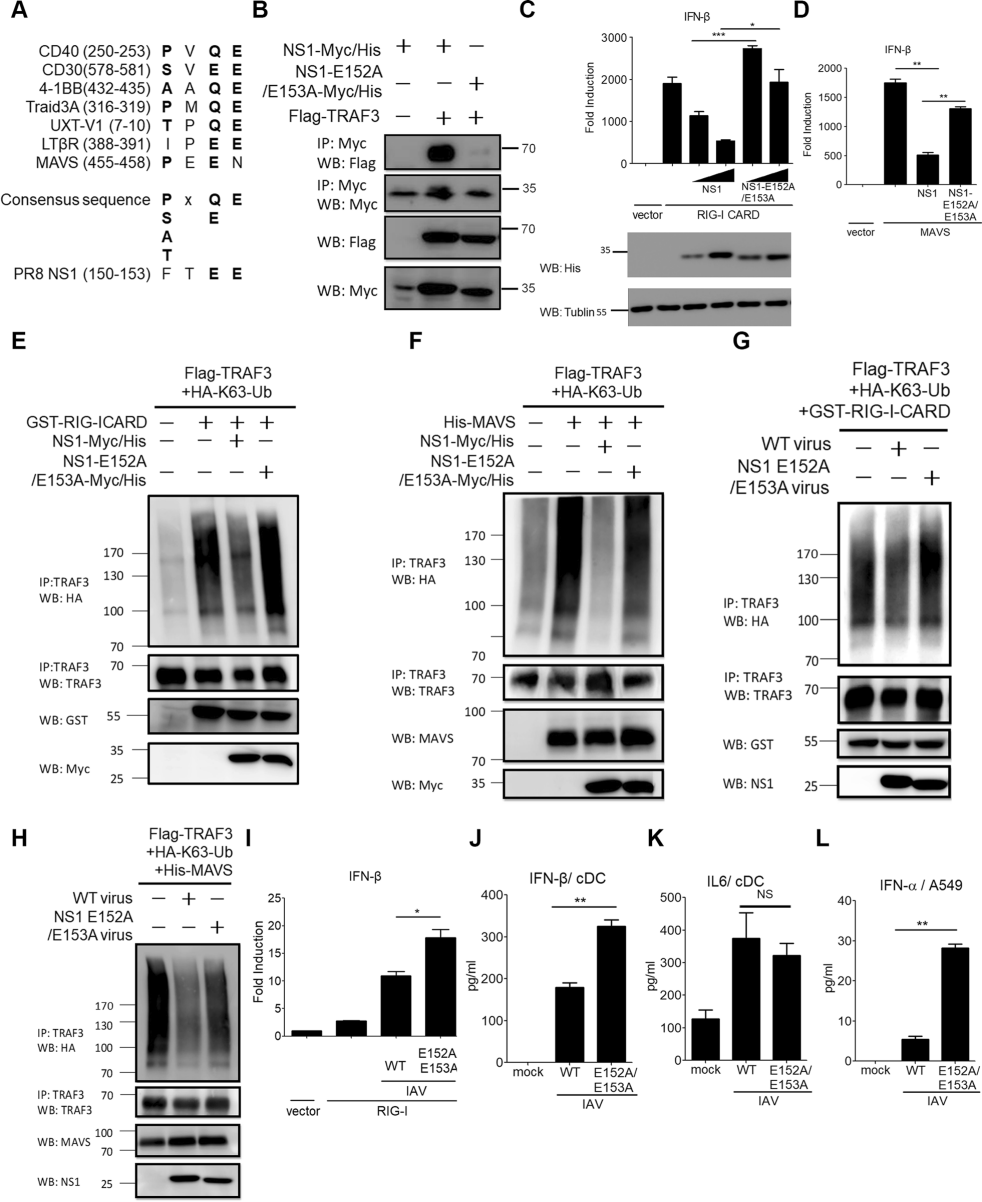
The NS1 E152/E153 residues are critical for targeting TRAF3 to impair RIG-I signaling to type I IFN induction. **A** The sequence alignment of several TRAF3-interacting motifs (TIMs) in known TRAF3-interacting proteins to reveal the TRAF3-interacting consensus motifs and a potential TIM in IAV NS1 (PR8). **B** HEK293T cells transfected with Flag-TRAF3 together with NS1-Myc/His or NS1 E152A/E153A-Myc/His were subjected to the IP-WB analysis. **C**, **D** Luciferase reporter assays were performed to assess the effect of NS1-E152A/E153A on IFN-β promoter activity induced by RIG-I-CARD (**C**) or MAVS (**D**). HEK293 cells were transfected with IFN-β-Luc together with control vector, RIG-I-CARD (**C**), or MAVS (**D**) plus two doses of NS1 and NS1 E152A/E153A, and transfected cells were subjected to analyze IFN-β promoter activity. **E, F** The effect of NS1 and NS1-E152A/E153A on TRAF3 K63-linked ubiquitination was analyzed by the IP-WB analysis. HEK293T cells were transfected with HA-K63 Ub and Flag-TRAF3 in the presence of GST-RIG-I-CARD (**E**) or MAVS (**F**) together with NS1-Myc/His or NS1 E152A/E153A-Myc/His as indicated. Cell lysates were first immunoprecipitated by anti-TRAF3 antibody and then immunoblotted by anti-HA antibody. **G, H** Like panels **E** and **F**, the IP-WB analysis was performed to assess TRAF3 K63-linked ubiquitination under virus infection of IAV wild type virus (WT PR8, 1 MOI) or NS1 E152A/E153A mutant virus (1 MOI). **I** Luciferase reporter assays were performed to assess RIG-I signaling to IFN-β promoter induction in the presence of IAV WT or NS1 E152A/E153A virus. HEK293 cells transfected with IFN-β-Luc and RIG-I were left untreated or infected with WT virus (1 MOI) or NS1 E152A/E153A virus (1 MOI), and the treated cells were harvested for analyzing the IFN-β promoter activity. **J, K** Bone marrow-derived conventional dendritic cells (cDCs) were infected with WT virus (10 MOI) or NS1 E152A/E153A virus (10 MOI) for 24 h, and the production of IFN-β (**J**) and IL-6 (**K**) was measured by ELISA. **L** A549 were infected with WT virus (1 MOI) or NS1 E152A/E153A virus (1 MOI) for 24 h, and the production of human IFN-α was measured by ELISA. Values represent the mean ± SD of triplicate samples (**C, D, I-L**). *P < 0.05, **P < 0.005, and NS stands for “not significant” (Student’s *t*-test). Data are representative of three experiments

In light of our biochemical findings aforementioned, we speculated that IAV NS1 might harbor a binding motif within the ED-C domain for targeting TRAF3. Cumulative evidence indicates that several signaling mediators, such as MAVS, CD40, and UXT-V1, interact with TRAF3 through a TRAF3-interacting motif (TIM) (S/T-x-Q/E-E) (Fig. 4A) [40, 51]. Through the sequence analysis, we identified a highly conserved FTEE motif (a. a. 150-153) in the NS1 proteins from the PR8 strain and other IAV strains (Fig. 4A and Additional file 1: Fig. S4A). We hypothesized that the FTEE motif in IAV NS1 might resemble a TRAF3-interacting motif to target TRAF3 to block the K63-linked ubiquitination and activation of TRAF3. To test this idea, we generated an NS1 mutant in which E152/E153 residues were changed to A152/A153, designated NS1-E152A/E153A, for our study. Co-IP results first showed that the NS1-E152A/E153A mutant, compared to NS1 wild type (WT), lost its ability to bind TRAF3 significantly (Fig. 4B). Further, the NS1-E152A/E153A mutant also alleviated the blocking effect on RIG-I CARD-induced IFN-β activation (Fig. 4 C, the upper panel). The protein levels of NS1 WT and the NS1-E152A/E153A mutant were comparable (Fig. 4C, the bottom panel). A similar result was also observed in MAVS-induced IFN-β activation (Fig. 4D). However, neither NS1 nor the NS1-E152A/E153A mutant caused the notable downregulation of RIG-I CARD-induced NF-κB activation (Additional file 1: Fig. S4B), further supporting the idea that NS1 selectively target the RIG-I-TRAF3-IFN-β axis via its ED. Next, we determined the effect of NS1 E152/E153 on the K63-linked ubiquitination of TRAF3 during RIG-I signaling. Our results revealed that the NS1-E152A/E153A mutant, compared to NS1 WT, substantially diminished the ability to block the K63-linked ubiquitination of TRAF3 induced by RIG-I-CARD and MAVS respectively (Fig. 4E, F). Together, our data suggest that NS1 E152/E153 residues are important for targeting TRAF3 during RIG-I signaling to type I IFN activation.

Next, we investigated the effect of NS1 E152/E153 on RIG-I signaling in the context of IAV infection. A recombinant IAV which carries the NS1-E152A/E153A mutant, designated NS1 E152A/153A virus, was generated for our experiments. Viral infection experiments demonstrated that the IAV WT virus, but not NS1 E152A/153A virus, effectively suppressed the K63-linked ubiquitination of TRAF3 induced by RIG-I-CARD and MAVS respectively (Fig. 4G, H). Accordingly, the NS1 E152A/E153A virus alleviated the suppression of RIG-I-mediated IFN-β activation in HEK293 cells (Fig. 4I). Given the critical role of RIG-I in fibroblasts and cDCs to trigger type I IFN production upon sensing IAV and other RNA virus infections [23, 24, 35], we assessed the effect of WT and NS1 E152A/E153A viruses on mouse bone marrow-derived cDCs. ELISA results showed that the NS1 E152A/153A virus induced higher IFN-β production from cDCs than the WT virus did (Fig. 4J), while both of them induced similar levels of IL-6 production from cDCs (Fig. 4K). Since A549 cells are a lung epithelial cell line commonly used for studying IAV infection, we also examine the effect of the NS1 E152A/153A virus on type I IFN induction in A549 cells. Likewise, the NS1 E152A/153A virus induced higher IFN-α in A549 cells (Fig. 4L). These data confirm the importance of NS1 E152 and E153 residues in targeting the RIG-I-TRAF3-IFN-β axis during IAV infection.

### The effect of NS1 E152/E153 residues on TLR3 and TLR7 signaling to type I IFN induction

**Fig. 5:**
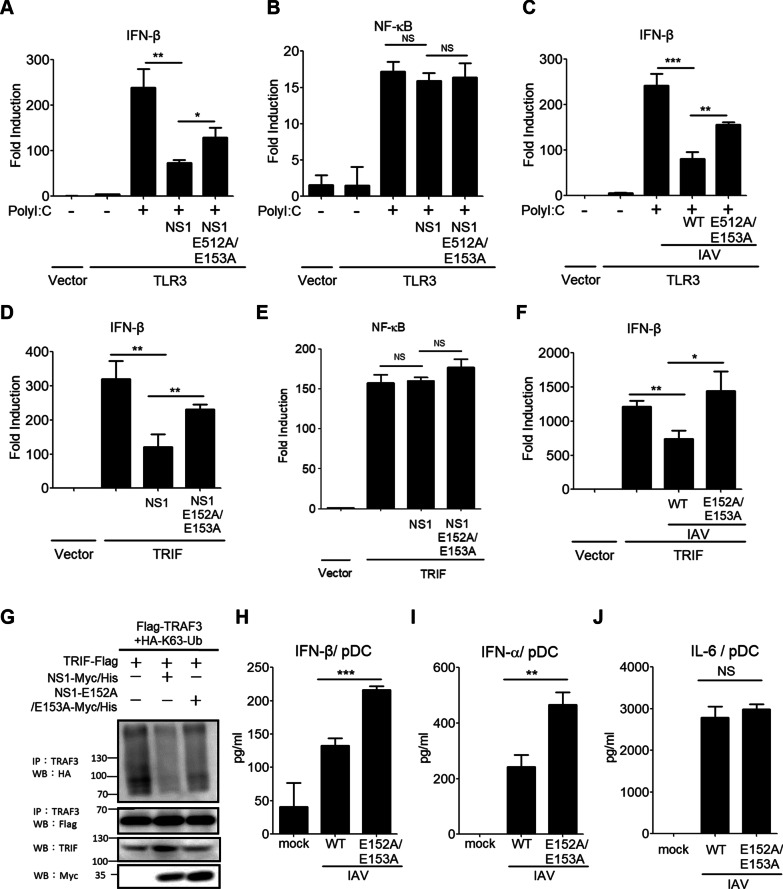
NS1 E152/E153 residues are critical for blocking TLR3 and TLR7 signaling to type I IFN induction. **A, B** HEK293 cells were transfected with IFN-β-Luc (**A**) or pELAM-Luc (**B**) together with control vector or TLR3 in combination with NS1 or NS1 E152A/E153A. After 24 h, transfected cells were left untreated or treated with poly I:C (1 µg/ml) (A) or poly I:C (20 µg/ml) (**B**). After another 16 h, treated cells were harvested for analyzing the IFN-β or NF-κB promoter activity. **C** HEK293 cells were transfected with IFN-β-Luc together with a control vector or TLR3. After 16 h, transfected cells were infected with WT virus (PR8, 0.2 MOI) or NS1 E152A/E153A virus (PR8, 0.2 MOI). After 12 h, transfected cells were left untreated or treated with poly I:C (1 µg/ml). After another 12 h, treated cells were harvested for analyzing the IFN-β promoter activity. **D, E** HEK293 cells were transfected with IFN-β-Luc (**D**) or pELAM-Luc (**E**) together with control vector or TRIF in combination with NS1 or NS1 E152A/E153A. After 16 h, cells were harvested for analyzing the IFN-β or NF-κB promoter activity. **F** HEK293 cells were transfected with IFN-β-Luc together with the control vector or TRIF. After 8 h, transfected cells were infected with WT virus (PR8, 0.2 MOI) or NS1 E152A/E153A virus (PR8, 0.2 MOI) for another 16 h. Cells were harvested for analyzing the IFN-β promoter activity. **G** HEK293T cells were transfected with HA-K63 Ub, Flag-TRAF3, and TRIF-Flag together with NS1-Myc/His or NS1 E152A/E153A-Myc/His as indicated. The cell lysates were subjected to the IP-WB analysis. **H-J** Bone marrow-derived plasmacytoid dendritic cells (pDCs) were infected by WT virus (PR8, 10 MOI) or NS1 E152A/E153A virus (PR8, 10 MOI) for 24 h, and the production of IFN-β (**H**), IFN-α (**I**), and IL-6 (**J**) was measured by ELISA. Values represent the mean ± SD of triplicate samples (**A-F, H-K**). *P < 0.05, **P < 0. 05, ***P < 0.0005, and NS stands for “not significant” (Student’s *t*-test). Data are representative of three experiments

In addition to the RLR pathways, TRAF3 is also involved in linking the TLR3 and TLR7 pathways to type I IFN production [50]. Although TLR3 and TLR7 are shown to detect IAV infection to trigger innate immune responses, it remains unclear if IAV NS1 might counteract the TLR3 and TLR7 pathways to type I IFN activation via targeting TRAF3. To this end, we first examined the effect of NS1 WT and the NS1-E152A/E153A mutant on TLR3-induced IFN-β promoter activation. Our results showed that NS1 WT impaired TLR3-mediated IFN-β promoter activation upon polyI:C stimulation whereas the NS1-E152A/E153A mutant reduced this blocking effect (Fig. 5A). However, neither NS1 WT nor its mutant was shown to block TLR3 signaling to the NF-κB promoter (Fig. 5B). Viral infection experiments also showed results similar to biochemical analyses (Fig. 5C and Additional file 1: Fig. S4C). Since Trif is a critical adaptor linking TLR3 signaling to the TRAF3-IFN-β axis, we next examined the effect of NS1 WT and E152A/E153A mutant on Trif signaling to the IFN-β promoter. Like the TLR3 scenario, we noticed that the NS1-E152A/E153A mutant alleviated this blocking effect (Fig. 5D). Neither NS1 WT nor NS1-E152A/E153A mutant blocked Trif signaling to the NF-κB promoter (Fig. 5E). Also, the WT virus but not NS1 E152A/E153A virus impaired Trif-induced IFN-β promoter activation (Fig. 5F). Biochemical analyses demonstrated that NS1 WT, but not the NS1-E152A/E153A mutant, effectively suppressed Trif-mediated TRAF3 K63-ubiquitination (Fig. 5G). These data demonstrate that the E152/E153 residues of NS1 are critical for targeting TRAF3 in the TLR3-Trif- IFN-β axis.

To investigate the effect of NS1 E152/E153 on TLR7 signaling to type I IFN induction, mouse bone marrow-derived pDCs were infected with WT virus and NS1 E152A/E153A virus, and then cytokine production was analyzed by ELISA. Our results revealed that the NS1 E152A/153A virus induced higher production of IFN-β, IFN-α, and RANTES from pDCs than the WT virus did (Fig. 5H, I, and Additional file 1: Fig. S4D), while both of WT and mutant viruses induced similar levels of IL-6 production from pDCs (Fig. 5J). These results indicate that the NS1 E152/E153 residues are crucial for IAV to counteract TLR7 signaling to type I IFN production.

### NS1 E152/E153 residues are critical for the in vivo pathogenicity of IAV infection

**Fig. 6:**
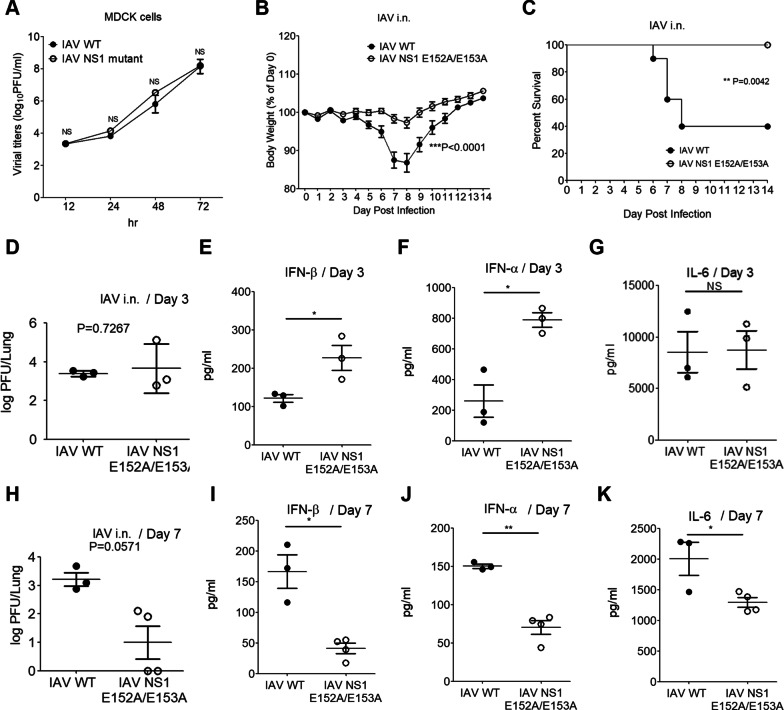
The NS1 E152/E153 residues confer the in vivo pathogenicity of IAV infection. **A** The growth curve of recombinant IAV WT virus (PR8) and NS1 E152A/E153A virus (PR8) in MDCK cells. Values represent the mean ± SD of duplicate samples. NS stands for “not significant” (Student’s *t*-test). Data are representative of three experiments. **B, C** Mice were infected with WT virus (50 PFU; n=10) or NS1 E152A/E153A virus (50 PFU; n=10) by intranasal (i.n.) infection. The percentage of weight loss (**B**) and survival rates (**C**) of mice were monitored daily for 14 days. The weight loss difference between the two groups of mice was statistically significant (***P < 0.0001 by two-way ANOVA). Values represent the mean ± SEM. Kaplan-Meier survival curves of the two groups were statistically significant (**P<0.01 by Log-rank (Mantel-Cox) Test). **D-G** Mice were infected with WT virus (1000 PFU; n=3) or NS1 E152A/E153A virus (1000 PFU; n=3) by i.n. infection. Lung viral titers of infected mice were measured on day 3 postinfection by plaque assay and analyzed by the Mann-Whitney U test (**D**). The production of IFN-β (**E**), IFN-α (**F**), and IL-6 (**G**) in lung homogenates was determined by ELISA. Values represent the mean ± SEM. *P < 0.05, and NS stands for “not significant” (Student’s *t*-test). **H-K** Mice were infected with WT virus (50 PFU; n=3) or NS1 E152A/E153A virus (50 PFU; n= 4) by i.n. infection. Like panels **D**-**G**, viral titers and cytokines were measured on day 7 postinfection by plaque assay and ELISA

Thus far our data demonstrated the importance of NS1 E152/E153 residues in subverting multiple RNA sensing pathways to type I IFN activation during IAV infection in transfected and primary cells. Further, we assessed the in vivo role of NS1 E152/E153 residues in viral replication and pathogenicity in mice. First, our replication analyses showed that IAV WT and NS1 E152A/153A viruses displayed similar replication rates in MDCK cells (Fig. 6A), suggesting that NS1 E152/E153 residues were not essential for IAV replication. In vivo studies showed that mice infected with the WT virus exhibited more significant weight loss in comparison to those infected with NS1 E152A/E153A virus (Fig. 6B and Additional file 1: Fig. S5A, B). Consequently, mice infected with NS1 E152A/E153A virus led to a better survival rate (Fig. 6C). Considering these data, we reasoned that infection of NS1 E152A/E153A virus might induce higher type I IFN production in mice, which in turn mounts better antiviral immunity against IAV infection. To test this idea, we assessed the viral titer and cytokine production in mouse lungs at the early (day 3 post-infection) and late (day 7 post-infection) stages of IAV infection. Mice infected with WT and NS1 E152A/E153A virus were sacrificed at day 3 post-infection for determining viral titers in the lungs. Plaque assays showed that WT and NS1 E152A/E153A viruses displayed similar viral loads in mouse lungs during the early infection (Fig. 6D). Of note, ELISA results revealed that NS1 E152A/E153A virus induced higher cytokine production of IFN-α, IFN-β, and RANTES in mouse lungs than WT virus did (Fig. 6E, F, and Additional file 1: Fig. S5C). In contrast, WT and NS1 E152A/E153A viruses induced similar levels of IL-6 production in mouse lungs (Fig. 6G). Similar approaches were used to measure the samples from infected mice at day 7 post-infection. As shown in Fig. 6H, the lung viral titer of NS1 E152A/E153A virus was notably lower than that of WT virus at the late stage of infection (Fig. 6H), implying that NS1 E152A/E153A virus might be under elimination by host immune responses while WT virus better evades from host immune responses to remain a higher titer. Likewise, higher cytokine production of type I IFNs and IL-6 was observed in WT virus-infected mice at day 7 post-infection (Fig. 6I-K and Additional file 1: Fig. S5D), suggesting a consequence of different viral titers of two viruses. Our data demonstrate that the NS1 E152/E153 residues are critical for IAV to suppress type I IFN-mediated antiviral responses and confer pathogenicity in vivo.

## Discussion

**Fig. 7:**
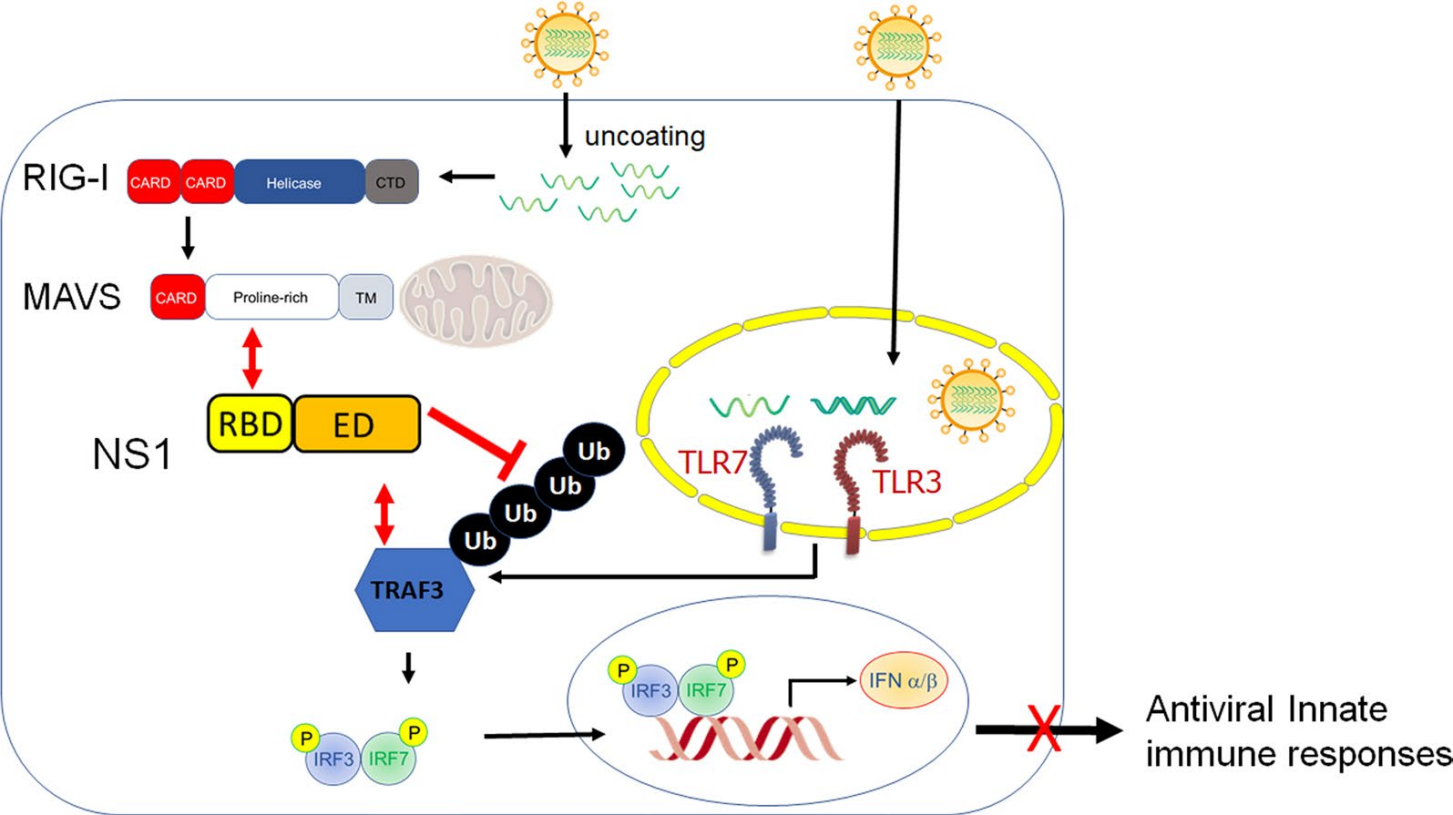
Model for IAV NS1-mediated immune evasion through targeting TRAF3 to impair the RIG-I, TLR3, and TLR7 pathways to type I IFN production. RIG-I, TLR3, and TLR7 are involved in detecting IAV RNA to trigger type I IFN-mediated antiviral immunity. IAV NS1 interacted with TRAF3 through its TRAF3-binding motif (TIM) in the C-terminal effector domain. This interaction blocked TRAF3 ubiquitination and activation, which is required for RIG-I, TLR3, and TLR7 pathways to type I IFN production

In the present study, we reveal a novel NS1-mediated immune evasion mechanism by which NS1 subverts the RIG-I, TLR3, and TLR7 pathways to type I IFN production through targeting an E3 ubiquitin ligase TRAF3, which is a common regulator linking these RNA sensing pathways to type I IFN production (Fig. 7). It is likely that RIG-I, TLR3, and TLR7 function in different cell types in response to IAV infection, and cooperatively provide a broad spectrum of protection against IAV infection in vivo. Given this idea, this novel NS1 targeting strategy renders IAV a greater advantage to thrive in the host leading to severe pathogenicity. Additionally, recent evidence illustrates the importance of type III IFNs in defending IAV infection in the respiratory tract [1]. It will be noteworthy to examine the impact of this NS1-TRAF3 targeting strategy on type III IFN production during IAV infection.

This work was initially conducted in our lab several years ago. Biochemical analyses of several NS1 truncated mutants led us to identify the distal C-terminal effector domain (a. a. 125-230) responsible for the TRAF domain of TRAF3 to inhibit the K63-linked ubiquitination of TRAF3, which in turn intercepts RIG-I signaling to type I IFN induction. These findings were first published in the author’s theses [33, 34]. In consistence with our early findings, a recent study reported that the NS1 protein from the IAV H5N1 strain targets TRAF3 via its ED to interfere with RIG-I signaling to type I IFN production [45]. Our continued efforts extended this NS1-TRAF3 targeting effect onto the TLR3 and TLR7 pathways and revealed the FTEE motif (a. a. 150-153) in the NS1 effector domain as a TRAF3-interacting motif (TIM). Although our current work demonstrated this novel NS1-mediated immune evasion in the context of IAV (H1N1, PR8 strain) infection, the FTEE motif is highly conserved in the NS1 proteins from several IAV strains including H1N1, H3N2, H5N1, and H7N9 (Additional file 1: Fig S4). Thus, it is likely that this NS1-TRAF3 targeting strategy might be commonly employed by different IAV strains to suppress type I IFN production to facilitate viral spread in the host. Future studies will be interesting to test this idea.

Our biochemical results pinpointed the E152/E153 residues of NS1 critical for targeting TRAF3 and then inhibiting its K63-linked ubiquitination. Consistently, functional analyses using a recombinant IAV carrying the NS1-E152A/E153A mutations confirmed the importance of the E152/E153 resides of NS1 in the suppression of type I IFN production and in the contribution to in vivo pathogenicity. To our best knowledge, there is no other known function attributed to the E152/E153 resides of NS1 before our current work. However, the contribution of other amino acids in the FTEE motif to TRAF3 interaction and NS1-mediated immune evasion remains unclear at present. Further studies are needed to address this issue, and it will be intriguing to test more combinatory mutations of the FTEE motif to affect the NS1-mediated immune evasion and IAV pathogenicity. Further structural analyses may provide mechanistic insights towards better understanding the NS1-TRAF3 interaction.

## Conclusions

In light of NS1-mediated countermeasure in type I IFN-mediated antiviral responses, NS1-modified IAV mutant strains have been developed as vaccine candidates, which have shown broad immune protection against both homologous and heterologous IAV challenges in several animal models [47]. Our findings shown here not only expand our understanding of NS1-mediated countermeasure and pathogenicity but also have potential implications in the development of attenuated IAV vaccines or antivirals in the future.

## Supplementary Information

**Additional file 1. Supplemental Figure 1**. The effect of IAV NS1 1-124 on blocking RIG-I signaling to IFN-β activation.** Supplemental Figure 2**. IAV NS1 is co-localized with TRAF3 in mammalian cells.** Supplemental Figure 3**. IAV NS1 uses its ED-C to block the K63-linked ubiquitination of TRAF3 during RIG-Isignaling.** Supplemental Figure 4**. IAV NS1 E152/E153 residues are not essential for blocking the NF-κB promoter activity during RIG-I, TLR3 or TLR7 signaling.** Supplemental Figure 5**. NS1 E152/E153 residues confer the in vivo pathogenicity of IAV infection.** Supplemental Table 1**. Primers used for RT-PCR, cloning and mutagenesis.** Supplemental Table 2**. Antibodies.

## Data Availability

The original contributions presented in the study are included in the article/supplementary material, further inquiries can be directed to the corresponding authors.

## Abbreviations

IAV: Influenza A virus
NS1: Non-structural protein 1
IFN: Type I interferon
ISGs: Interferon-stimulated genes
cDCs: Conventional dendritic cells
pDCs: Plasmacytoid dendritic cells
ssRNA: Single-stranded RNA
RBD: RNA-binding domain
ED: Effector domain
MDCK: Madin-Darby canine kidney cells
CCS: Cosmic calf serum
BMDCs: Bone marrow-derived dendritic cells
Co-IP: Co-immunoprecipitation
WB: Western blot
PFA: Paraformaldehyde
DAPI: 4’,6-Diamidino-2-phenylindole
SEM: Standard error of the mean
SPF: Specific pathogen-free
TIM: TRAF3-interaction motif
IF: Immunofluorescence
WT: Wild type
ELISA: Enzyme-linked immunosorbent assay
