# Supplementary material for: Influenza a virus NS1 resembles a TRAF3-interacting motif to target the RNA sensing-TRAF3-type I IFN axis and impair antiviral innate immunity
Source: J Biomed Sci. 2021 Oct 5;28:66. doi: 10.1186/s12929-021-00764-0 (PMC8491413; doi:10.1186/s12929-021-00764-0)
Supplement: Supplementary file 1 — Additional file 1. Supplemental Figure 1. The effect of IAV NS1 1-124 on blocking RIG-I signaling to IFN-β activation. Supplemental Figure 2. IAV NS1 is co-localized with TRAF3 in mammalian cells. Supplemental Figure 3. IAV NS1 uses its ED-C to block the K63-linked ubiquitination of TRAF3 during RIG-Isignaling. Supplemental Figure 4. IAV NS1 E152/E153 residues are not essential for blocking the NF-κB promoter activity during RIG-I, TLR3 or TLR7 signaling. Supplemental Figure 5. NS1 E152/E153 residues confer the in vivo pathogenicity of IAV infection. Supplemental Table 1. Primers used for RT-PCR, cloning and mutagenesis. Supplemental Table 2. Antibodies. [file 12929_2021_764_MOESM1_ESM.docx]

Additional file 1

**
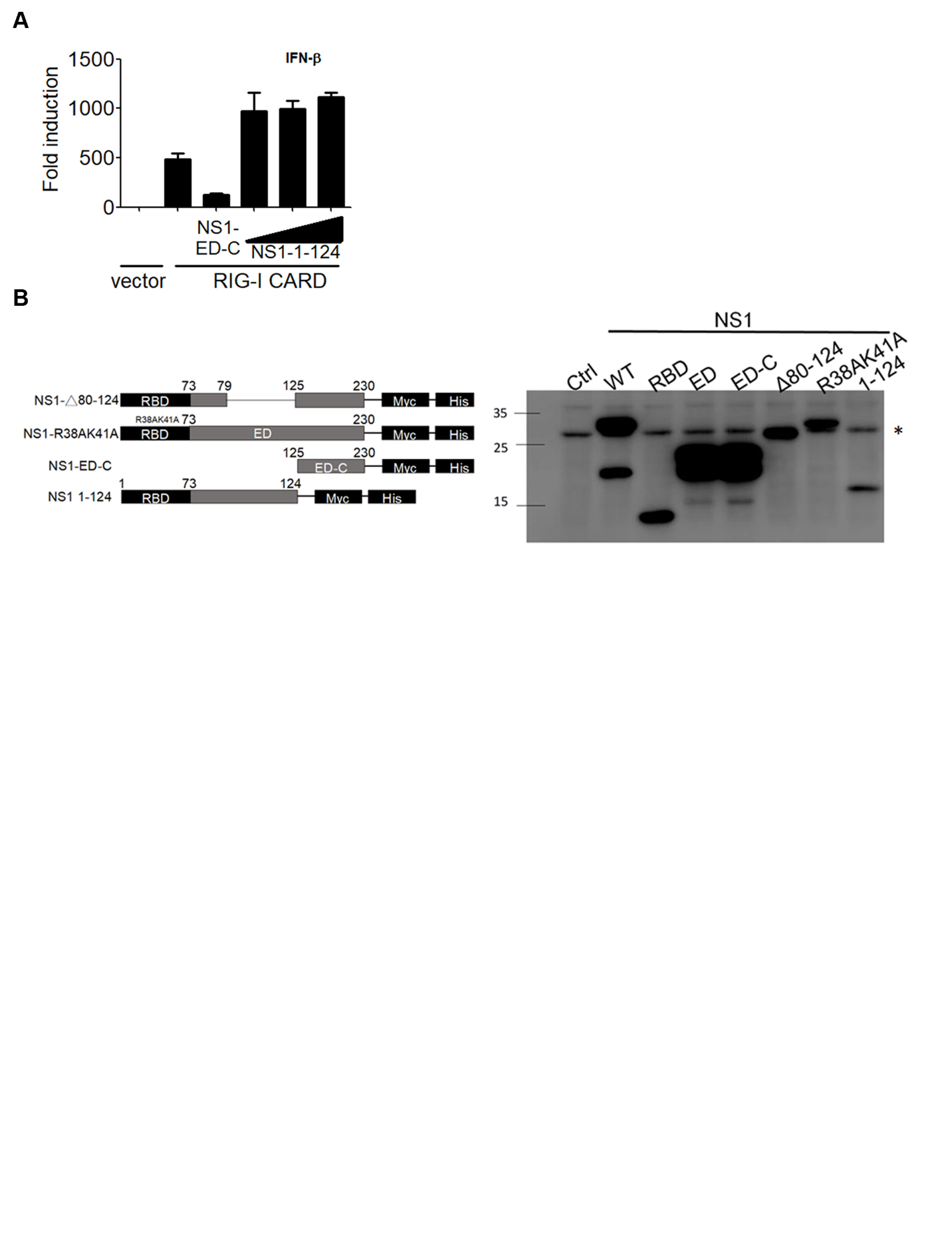
**

**Additional file 1: Figure 1** The effect of IAV NS1 1-124 on blocking RIG-I signaling to IFN-β activation. (A) HEK293 cells were transfected with IFN-β-Luc together with control vector, RIG-I CARD, and NS1-ED-C or increasing amounts of NS1-1-124 as indicated for analyzing IFN-β luciferase activity. (B) Schematic structures of IAV NS1 and its truncated mutants with C-terminal tagged Myc/His (left). The expression of these constructs in HEK293T cells was examined by Western blot (right). * indicated the non-specific band recognized by the anti-Myc antibody. Values represent the mean ± SD of duplicate samples (A). Data are representative of three experiments, and **is p<0.005 by unpaired t-test.

**
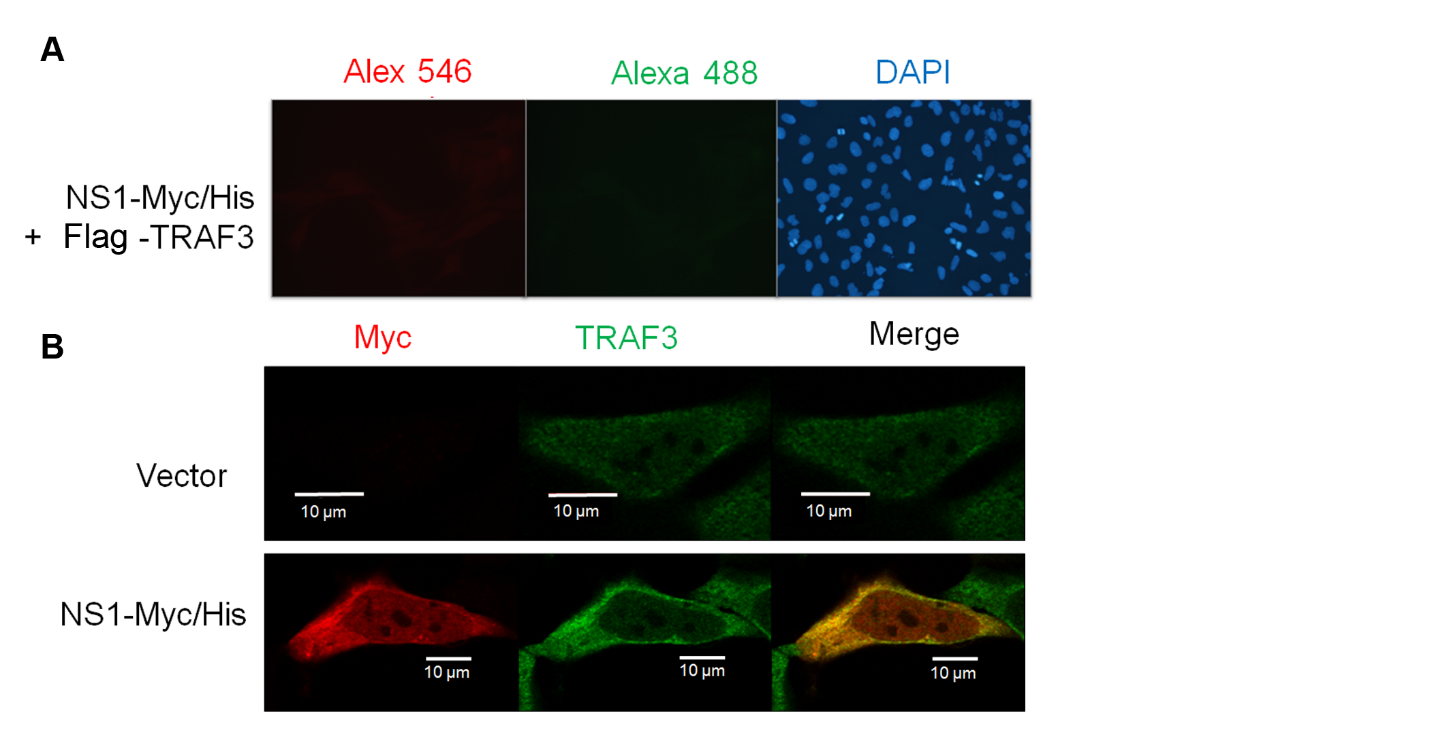
**

**Additional file 1:Figure 2** IAV NS1 is co-localized with TRAF3 in mammalian cells. (A) HEK293 cells were transfected with NS1-Myc/His together Flag-TRAF3 encoding plasmid for 24 h. Then the cells were fixed and immunostained with anti-mouse IgG-Alex 546 and anti-rabbit IgG-Alexa 488. (B) HEK293 cells were transfected with control vector or NS1-Myc/His for 24 h. Then the cells were fixed and immunostained with anti-Myc and anti-TRAF3 antibodies. Then the cells were subjected to confocal microscopy.


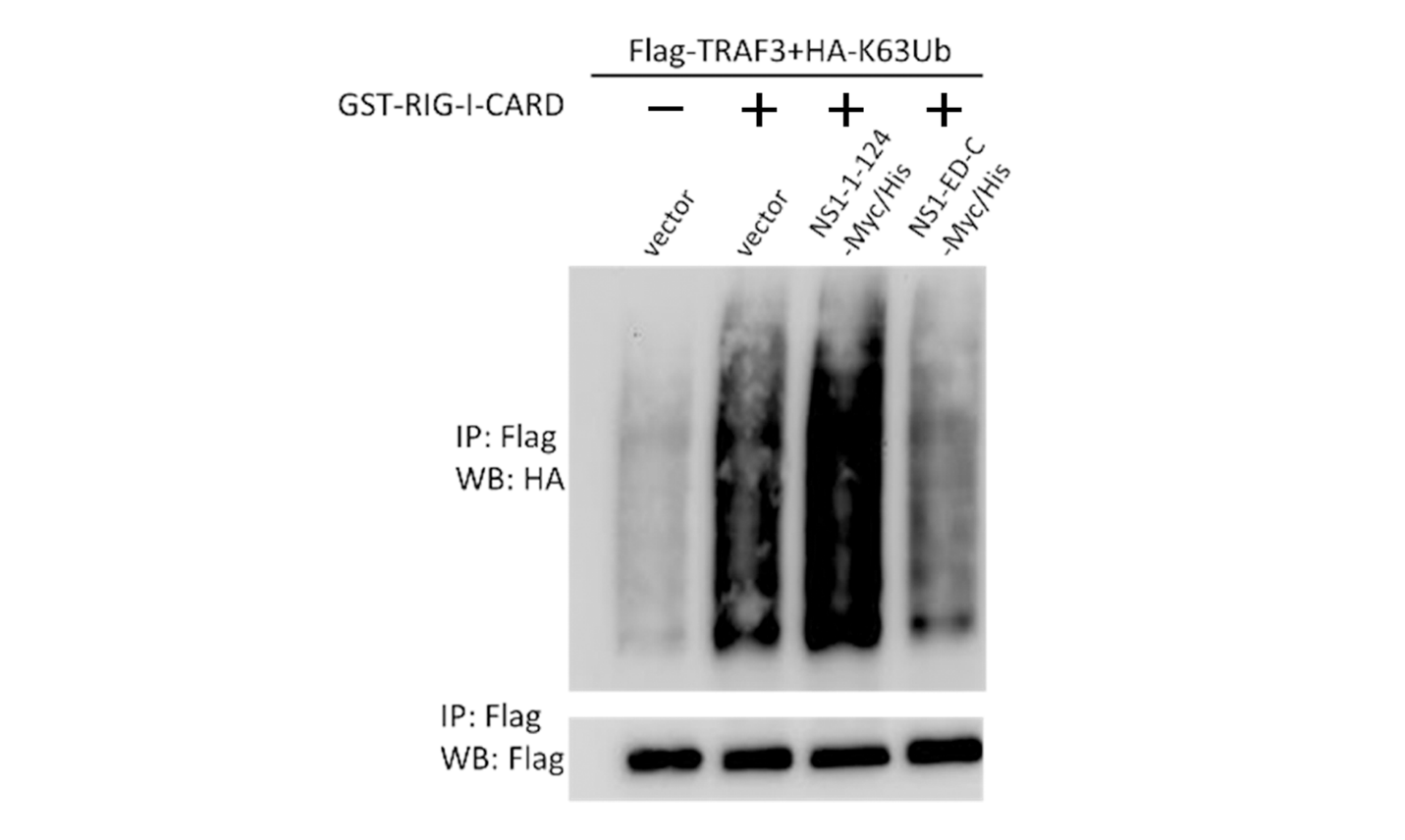


**Additional file 1:Figure 3** IAV NS1 uses its ED-C to block the K63-linked ubiquitination of TRAF3 during RIG-I signaling. HEK293T cells were transfected HA-tagged K63 Ub, Flag-tagged TRAF3, and RIG-I CARD together with NS1-1-124 or NS1-ED-C constructs. Cell lysates were subjected to the IP-WB analysis.

**
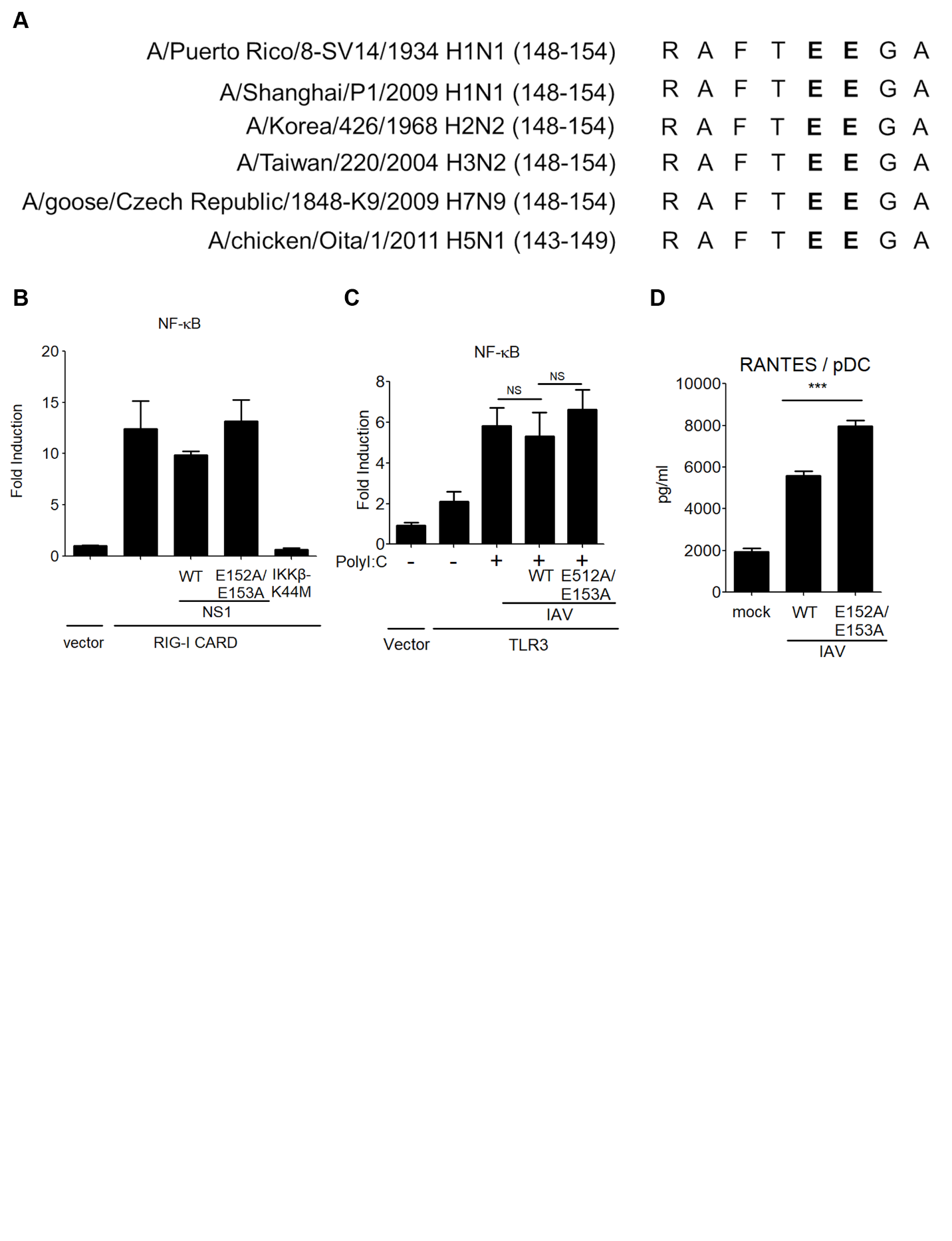
**

**Additional file 1:Figure 4** IAV NS1 E152/E153 residues are not essential for blocking the NF-κB promoter activity during RIG-I, TLR3 or TLR7 signaling. (A) The amino acid sequence alignment of a potential TRAF3-interacting motif in the NS1 proteins from various IAV strains. (B) HEK293 cells were transfected with pELAM-Luc together with control vector or RIG-I CARD in combination with NS1, NS1 E152A/E153A, or an IKKβ inactive mutant. After 24 h, the cells were harvested for analyzing the NF-κB promoter activity. (C) HEK293 cells were transfected with pELAM-Luc together with a control vector or TLR3. After 16 h, transfected cells were infected with wild-type virus (IAV/PR8, 0.2 MOI) or NS1 E152A/E153A virus (IAV/PR8, 0.2 MOI). After 12 h, transfected cells were left untreated or treated with poly I:C (1 µg/ml). After another 12 h, treated cells were harvested for analyzing the NF-κB promoter activity. Values represent the mean ± SEM of triplicate samples (B and C). NS stands for “not significant” (Student’s *t*-test). Data are from one experiment representative of three. (D) Bone marrow-derived plasmacytoid dendritic cells (pDCs) were infected by WT virus (PR8, 10 MOI) or NS1 E152A/E153A virus (PR8, 10 MOI) for 24 hours, and the production of RANTES was measured by ELISA. Values represent the mean ± SEM of triplicate samples. **P* < 0.05, ***P* < 0. 05, ****P* < 0.0005, and NS stands for “not significant” (Student’s *t*-test). Data are representative of three experiments.

**
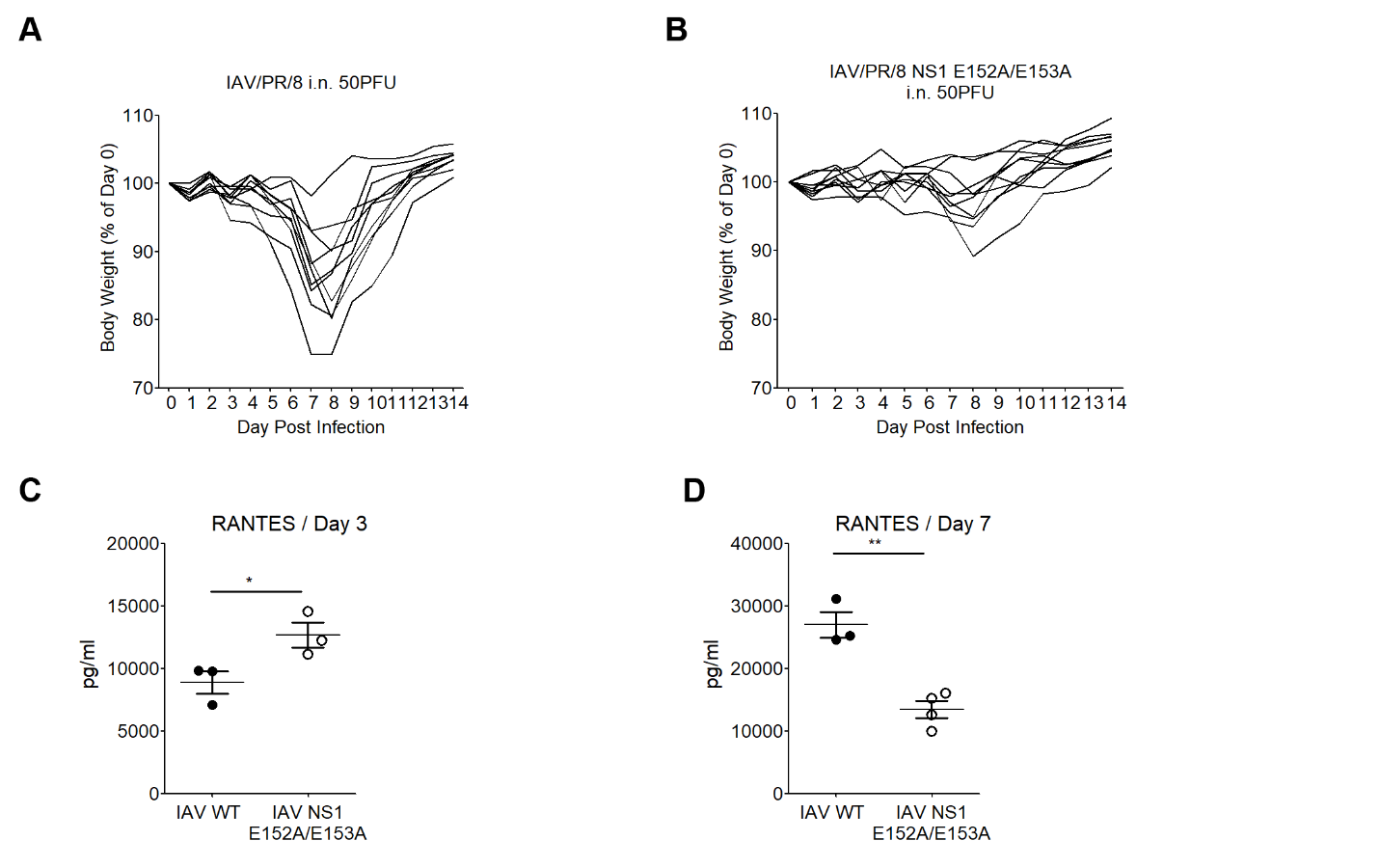
**

**Additional file 1:Figure 5** NS1 E152/E153 residues confer the in vivo pathogenicity of IAV infection. Related to Figure 6. (A, B) Mice were infected with wild-type virus (IAV/PR8) or NS1 E152A/E153A virus (IAV/PR8) (50 PFU; n=10 per group; approximately 6-7-weeks-of-age) by intranasal (i.n.) infection. Individual weight loss in mice was monitored daily for 14 days. (C) Mice were infected with IAV wild-type virus (IAV/PR8) or NS1 E152A/E153A virus (IAV/PR8) (1000 PFU; n=3 per group; approximately 6-7-weeks-of-age) by intranasal (i.n.) infection. The tissue viral loads were analyzed by the Mann-Whitney U test. The production of RANTES in lung homogenates was determined by ELISA. *is p<0.05 by unpaired t-test. (D) Mice were infected with IAV wild-type virus (IAV/PR8) or NS1 E152A/E153A virus (IAV/PR8) (50 PFU; n=3 or 4 per group; approximately 6-7-weeks-of-age) by intranasal (i.n.) infection. The production of RANTES in lung homogenates was determined by ELISA. Values represent the mean ± SEM. *is p<0.05 and **is p<0.005 by unpaired t-test.

**Additional file 1:Table 1.** Primers used for RT-PCR, cloning and mutagenesis.

| **Primer** | **Sequence** |
| --- | --- |
| PR8 NA | F: 5′- TTG GTC AGC AAG TGC ATG TC-3′  R: 5′- ACA GCC ACT GCT CCA TTA TC-3′ |
| 3X-Flag-RIG-I-CARD | F: 5′- AAGCTTACCACCGAGCAGCGA -3′  R: 5′- GGTACCTCATGGACATGAATTCTCACT -3′ |
| 3X-Flag-MAVS | F: 5′-AAGCTTACATTTGCTGAGGACAAG-3′  R: 5′- TCTAGATCACTGGGCCAGGCG -3′ |
| pEBG-RIG-I-CARD | F: 5′- GTTACCACCACCGAGCAGCGA - 3′  R: 5′- GCGGCCGCTCATGGACATGAATTCTC - 3′ |
| GFP-MAVS | F: 5′- CCC GGG A ACA TTT GCT GAG GAC - 3′  R: 5′- TCT AGA TCA CTG GGC CAG GCG - 3′ |
| NS1- Myc-His | F: 5′- GAATTCATGGATCCAAACACTGTG -3′  R: 5′- CTCGAGAACTTCTGACCTAATTGT -3′ |
| NS1- RBD- Myc-His | F: 5′- GAATTCATGGATCCAAACACTGTG -3′  R: 5′- CTCGAGGGATTCTTCTTTCAGAAT -3′ |
| NS1- ED- Myc-His | F: 5′- GAATTCATGGATGAGGCACTTAAA -3′  R: 5′- CTCGAGAACTTCTGACCTAATTGT-3′ |
| NS1- 1-124-Myc-His | F: 5′- GATGAGGCACTTAAAATGGATAAGAACATCATACTG -3′  R: 5′- CAGTATGATGTTCTTATCCATTTTAAGTGCCTCATC-3′ |
| NS1-R38A K41A- Myc-His | F: 5′- GATCGGCTTCGCGCAGATCAGGCATCCCTAAGAGGA-3′  R: 5′- TCCTCTTAGGGATGCCTGATCTGCGCGAAGCCGATC-3′ |
| NS1- ED-C- Myc-His | F: 5′- AGTGTGGTGGAATTCATGGATAAGAACATCATACTG-3′  R: 5′- CAGTATGATGTTCTTATCCATGAATTCCACCACACTC-3′ |
| NS1-1-124- Myc-His | F: 5′- ATGGACCAGGCGATCATGTGAAGAAATAAGATGGTT-3′  R: 5′- AACCATCTTATTTCTTCACATGATCGCCTGGTCCAT |
| NS1E152A/E153A-Myc-His | F: 5′- CTAAGGGCTTTCACCGCAGCGGGAGCAATTGTTGGC -3′  R: 5′- GCCAACAATTGCTCCCGCTGCGGTGAAAGCCCTTAG -3′ |
| NS1 E96A/E97A-Myc-His | F: 5′-CTAACTGACATGACTCTTGCGGCAATGTCAAGGGACTGGTCC -3′  R: 5′- GGACCAGTCCCTTGACATTGCCGCAAGAGTCATGTC AGTTAG -3′ |

**Additional file 1: Table 2.** Antibodies

| Antibodies | Supplier | Cat.number or Clone | Dilution condition for confocal microscopy |
| --- | --- | --- | --- |
| Mouse Anti-Myc | Millipore | Clone 4A6, #05-724 |  |
| Rabbit Anti-Myc | Sigma | C3956 | 10 µg/ml |
| Mouse Anti-Flag | Sigma | Clone M2, F1804 |  |
| Rabbit Anti-Flag | Sigma | F7425 |  |
| Anti-Influenza A NS1 | Santa Cruz | SC-130568 | 200 µg/ml |
| Rabbit Anti-TRAF3 | Santa Cruz | H-20, sc-948 | 1:100 |
| Mouse Anti-TRAF3 | Santa Cruz | G-6, sc-6933 |  |
| Rabbit Anti-MAVS | Santa Cruz | SC-68881 |  |
| Rabbit Anti-MAVS | Cell Signaling | #4983 |  |
| Rabbit anti-K63 linkage Specific polyubiquitin | Cell Signaling | Clone D7A11, #5621 |  |
| Mouse Anti-HA | Sigma | Clone HA-7, H9658 |  |
| Mouse Anti-actin | Millipore | MAB1501 |  |
| Donkey Anti-rabbit IgG (H+L) (Alexa Fluor® 488) | Abcam | ab150073 | 1:800 |
| Donkey Anti-rabbit IgG (H+L) (Alexa Fluor® 568) | Abcam | ab175470 | 1:500 |
